# Utility of nonlinear analysis of heart rate variability in early detection of metabolic syndrome

**DOI:** 10.3389/fphys.2025.1597314

**Published:** 2025-06-24

**Authors:** José Alberto Zamora-Justo, Myriam Campos-Aguilar, María del Carmen Beas-Jara, Pedro Galván-Fernández, Alberto Ponciano-Gómez, Santiago Cristóbal Sigrist-Flores, Rafael Jiménez-Flores, Alejandro Muñoz-Diosdado

**Affiliations:** ^1^ Unidad Profesional Interdisciplinaria de Biotecnología del Instituto Politécnico Nacional, Mexico City, Mexico; ^2^ Laboratorio de Inmunología, Unidad de Morfología y Función (UMF), Facultad de Estudios Superiores Iztacala, Universidad Nacional Autónoma de México, Tlalnepantla, Mexico; ^3^ Laboratorio de Fisiología del Esfuerzo (GIN), Unidad Interdisciplinaria de Investigación en Ciencias de la Salud y Educación (UIICSE), Facultad de Estudios Superiores Iztacala, Universidad Nacional Autónoma de México, Tlalnepantla, Mexico

**Keywords:** heart rate variability time series, nonlinear dynamic techniques, sample entropy, detrended fluctuation analysis, autonomic dysfunction

## Abstract

**Introduction:**

Metabolic syndrome (MetS) is a clinical condition characterized by multiple risk factors that significantly increase the likelihood of developing cardiovascular diseases and type 2 diabetes. Traditional markers, such as body mass index (BMI) and waist circumference, often fail to detect early metabolic dysfunctions.

**Methods:**

This study evaluated nonlinear characteristics of heart rate variability (HRV) series, including sample entropy (SampEn), multifractal spectrum parameters, and detrended fluctuation analysis (DFA). A total of 278 participants were classified into three groups: no metabolic alterations, one or two alterations, and MetS (defined as three or more alterations based on ATP III criteria). HRV data were recorded at three time points: rest, exercise, and recovery.

**Results:**

Participants with MetS showed significantly lower SampEn and DFA values at rest compared to those without alterations, indicating reduced signal complexity. Moreover, a decrease in SampEn was observed in individuals with one or two metabolic alterations, suggesting that autonomic dysfunction may begin in the early stages of metabolic risk.

**Discussion:**

These findings support the integration of nonlinear HRV analysis with traditional methods to improve the early detection and management of metabolic syndrome. The progressive reduction in heart rate signal complexity may serve as a sensitive marker of early autonomic dysfunction in metabolic deterioration.

## 1 Introduction

Metabolic syndrome (MetS) is a complex clinical condition characterized by the coexistence of multiple risk factors, including abdominal obesity, hypertension, dyslipidemia, and hyperglycemia. These factors significantly increase the likelihood of developing cardiovascular diseases and type 2 diabetes according to the Third Adult Treatment Panel (ATP III) of the National Cholesterol Education Program (NCEP) Over the past decades, its prevalence has risen alarmingly, affecting not only adults but also younger populations, highlighting the urgent need for more effective diagnostic and preventive strategies (Fahed et al., 2022; US Department of Health and Human Services et al., 2001; Murguía-Romero et al., 2015).

Traditional methods, such as body mass index (BMI) and waist circumference, are widely used to assess metabolic risk due to their simplicity and low cost. However, these tools have important limitations, particularly in young populations or individuals with metabolically healthy obesity. These traditional markers often fail to detect underlying metabolic alterations in seemingly healthy individuals, potentially delaying diagnosis and increasing the risk of severe long-term complications (Murguía-Romero et al., 2013; 2012).

In this context, the analysis of heart rate variability time series obtained from electrocardiographic (ECG) signals has emerged as a promising approach to complement traditional methods. Nonlinear parameters, obtained from the analysis of heart rate variability time series such as sample entropy and detrended fluctuation analysis (DFA), provide a detailed evaluation of cardiovascular dynamics that cannot be captured through conventional linear methods. These nonlinear analyses assess the complexity and stability of heart rhythms, offering a more precise insight into autonomic regulation and the adaptive capacity of the organism (Aguilar-Molina et al., 2019; Asgharzadeh-Bonab et al., 2020; Zhao et al., 2018).

Sample entropy quantifies the variability and irregularity of heart rhythms, while DFA identifies long-term correlations in time series. Previous studies have demonstrated that a reduction in the complexity of these signals is associated with a diminished ability of the cardiovascular system to adapt to physiological changes. This reduction in complexity could represent an early marker of autonomic dysfunction and an indicator of incipient metabolic risk (Horie et al., 2018; Rojas-Jiménez et al., 2021; Zhao et al., 2018). Several studies have also demonstrated that autonomic function, assessed through heart rate variability, is closely linked to metabolic processes such as fat oxidation and metabolic flexibility, indicating that reductions in signal complexity may reflect early impairments in energy regulation and substrate utilization (La Rovere et al., 2020; Monferrer-Marín et al., 2024; Porta et al., 2007). Moreover, these tools not only complement traditional markers but also have the potential to detect subtle metabolic alterations before clinical symptoms become evident (Horie et al., 2018; Muñoz-Diosdado et al., 2023).

Therefore, this study aims to evaluate whether the integration of nonlinear parameters derived from ECG analysis can improve the detection of MetS when combined with traditional markers. This approach seeks to provide a more comprehensive assessment of metabolic and autonomic health, enabling earlier and more personalized interventions to prevent progression to severe chronic conditions.

## 2 Materials and methods

### 2.1 Participant selection

Participants in the study were recruited voluntarily from three key locations: the Immunology Laboratory at the Morphology and Function Unit of the Faculty of Higher Studies Iztacala, UNAM; the Exercise Physiology Laboratory at UICSE, Faculty of Higher Studies Iztacala, UNAM; and the Laboratorio de Complejidad y Análisis de Señales of the Unidad Profesional Interdisciplinaria de Biotecnología del Instituto Politécnico Nacional. Individuals aged between 18 and 65 years, regardless of gender, were included if they met the following inclusion criteria: being in generally good health, without prior diagnoses of cardiovascular, metabolic, or neurological diseases, and not under treatment with medications that could affect cardiovascular or metabolic function.

Exclusion criteria included the presence of any condition that could affect the validity of the measurements, such as the consumption of stimulants (caffeine, tobacco, etc.) in the 24 h prior to the study, intense physical exercise in the 48 h before the measurements, or the use of medications that could alter autonomic or metabolic function. Individuals with a diagnosis of type 1 diabetes, uncontrolled hypertension, or any other chronic condition that could interfere with the interpretation of results were also excluded.

All subjects were thoroughly informed about the study’s objectives and procedures and signed an informed consent form before participating. The project was approved by the Ethics Committee of the Faculty of Higher Studies Iztacala, UNAM, with approval number CE/FESI/022020/1348.

### 2.2 Sample collection procedure

Samples were collected in three distinct phases to ensure the consistency and validity of the data. First, standard anthropometric measurements were performed on the participants, including weight, height, body mass index (BMI), and waist circumference. These measurements were taken following protocols established by the World Health Organization (WHO), using calibrated equipment and under controlled conditions to minimize variability between measurements. Participants were weighed without shoes and in light clothing, and waist circumference was measured at the level of the navel at the end of a gentle exhalation.

The second phase involved recording ECG signals using Fukuda Denshi FM-180 Holter monitor, which include built-in band-pass filter ranging from 0.05 to 40 Hz, helping to maintain signal stability even during physical activity. First, recordings were taken at rest for 30 min, followed by 30 min of physical activity (3.5 mph for young adults aged 18 to 30, and 3.0 mph for adults aged 30–65), these speeds were selected because they did not pose any health risks, considering that some participants were sedentary. No specific dietary restrictions were imposed prior to this phase, as the aim was to assess autonomic responses under habitual physiological conditions. This was followed by a 15-min recovery phase.

Similarly, for the biochemical evaluation, no dietary standardization was implemented prior to the fasting blood draw. This decision was made to reflect each participant’s general health and metabolic status under real-life conditions, rather than to assess the effect of a controlled intake. Therefore, no direct correspondence was expected between biochemical values and autonomic parameters recorded on a different day.

Finally, third phase consisted of the collection of blood samples after a minimum of 8 h of fasting to assess levels of glucose, lipids (total cholesterol, HDL, LDL, triglycerides), insulin, uric acid, and other relevant metabolic markers. Blood samples were collected by trained personnel using standardized venipuncture techniques. The samples were centrifuged and stored at 4°C–6°C until they were processed to prevent analyte degradation. Blood sample analysis was conducted at Grupo Diagnóstico Médico Proa S. A. de C. V. using automated equipment and certified reagents to ensure the accuracy and reproducibility of the results.

### 2.3 Electrocardiographic monitoring

Electrocardiographic monitoring was performed using a Holter Recorder Digital Walk FM-180 by Fukuda Denshi, which allowed continuous recording of the heart’s electrical activity during the different phases of the study. First, participants were fitted with the Holter and allowed to rest for 30 min to obtain a baseline of cardiac activity at rest. This initial rest period was crucial for establishing a reference point before subjecting participants to physical activity.

Next, participants engaged in a 30-min walk on a linear treadmill at the speeds mentioned above, during which the Holter continuously recorded the electrocardiographic signal (ECG), enabling the capture of data during moderate physical exertion. Once the walk was completed, participants were again placed at rest for 15 min, during which the Holter monitoring continued to assess cardiac recovery and heart rate dynamics following physical exertion.

In addition to ECG monitoring, participants’ blood pressure was measured at two key moments: during the initial rest period, and at the end of the post-exercise recovery period. These blood pressure measurements were taken using digital blood pressure monitors (HEM-7120-LA, OMRON Healthcare, INC.). All stages of electrocardiographic monitoring and blood pressure measurements were conducted at the three study sites under controlled conditions to minimize external variations that could affect the results.

### 2.4 Electrocardiographic signal analysis

The heart rate variability signal analysis was conducted using a combination of advanced techniques to assess the complexity and variability of the cardiac signal and to identify patterns associated with autonomic regulation. The data obtained from the Holter were processed and analyzed in several stages, focusing on detecting irregularities that could indicate metabolic and autonomic dysfunctions in the participants.

### 2.5 Data preprocessing

The ECG signal and heart rate variability (HRV) series were extracted from Holter software SCM-510, which calculates the positions of the R-peaks of the ECG to construct the RR interval series. It is important to note that no additional digital filtering was applied to the ECG signal since the built-in band-pass filter (0.05–40 Hz) in the Holter monitor effectively reduces baseline drift and high-frequency noise, ensuring signal quality even during physical activity. On the other hand, artifacts from HRV series were removed using a thresholding approach, since R-peak detection can sometimes fail—either by missing actual peaks or by mistakenly identifying noise as R-peaks. To address this, interbeat intervals greater than 1.5 s and less than 0.2 s were excluded, as such values typically result from the aforementioned detection errors. The complete interbeat interval signal was divided into three subseries: rest, exercise, and recovery. Each segment was defined based on the time associated with its corresponding stage; hence, 4,096 samples were taken during the resting period, and 2,048 samples during exercise. For the recovery phase, the number of samples varied depending on how long each subject took to return to their baseline heart rate.

It is worth mentioning that both the initial transition periods and the final stages of each test were excluded from all subseries, this ensured that only stable physiological states were analyzed, allowing the data to reflect consistent and reliable patterns. To further support the temporal stability of the selected segments, the coefficient of variation (CV) of the RR intervals was calculated. While CV is not a fully robust metric for nonlinear and non-stationary time series, it provides a useful reference when applied to short segments. The results showed that CV remained below 13% in all analyzed stages: during rest, CV was 9.44% ± 3.60%; during exercise, 4.14% ± 2.48%; and during recovery, 12.29% ± 3.84%. In contrast, the CV of the full-length series was 22.41% ± 6.36%, due to the high variability in heart rate throughout the entire recording period. This analysis also reveals a loss of heart rate variability during physical activity compared to variability at rest.

### 2.6 Entropy calculation

The entropy of the ECG signals was calculated using the sample entropy algorithm, which allows the assessment of signal complexity at different time scales. This approach is particularly useful for identifying irregularity and variability in cardiac signals, providing a measure of the dynamic complexity of the cardiovascular system (Aguilar-Molina et al., 2019). For its calculation it was used the following algorithm (Richman and Moorman, 2000):

Let *x* be the dataset of length *N* and the vector $x_{i}$ a subset of *x* with length *m*, from this it can be formed the vector $x_{j}=\left\{x_{i},x_{i+1},x_{i+2},\cdots ,x_{m}\right\}$ . The vectors $x_{i}$ and $x_{j}$ are similar if they comply that $\left|x_{i}-x_{j}\right|<r$ , where r=0.2σ , and σ is the standard deviation of *x*. Hence, the number of vectors that fulfil this fact is calculated using Equations 1, 2:

$$n_{i}^{m}=\sum_{\begin{array}{c} j=1\\ i\neq j \end{array}}^{N-m}\delta \left(i,j,m,r\right)$$ (1)

where:

$$\delta \left(i,j,m,r\right)=\left.\left\{\begin{array}{c} 1\;if\;\left|x_{i}-x_{j}\right|\;<\;r\\ \;\;\;\;0\;otherwise \end{array}\right.\right.$$ (2)

The similarity $A_{i}^{m}$ of the vectors is obtained from:

$$A_{i}^{m}=\frac{1}{N-m}n_{i}^{m}$$ (3)

Then using Equation 3, the average similarity is calculated by:

$$A^{m}=\frac{1}{N-m}\cdot \sum_{i=1}^{N-m}A_{i}^{m}$$ (4)

Using the same analysis, the average similarity (Equation 4) must be calculated from $x_{i}$ and $x_{j}$ vectors of length $m+1\;i.e.\;A^{m+1}$ . Finally, the sample entropy (E) was obtained from:

$$E=-\ln\left(\frac{A^{m+1}}{A^{m}}\right)$$ (5)

The sample entropy (Equation 5) was evaluated at rest, during exercise, and during recovery, allowing for comparison of autonomic responses in different physiological states (Solís-Montufar et al., 2020).

### 2.7 Detrended fluctuation analysis (DFA)

Detrended Fluctuation Analysis (DFA) was used to investigate the presence of long-term correlations in heart rate time series. This method is useful for detecting stability and fractal relationships in the cardiac signal, which can reflect the integrity of the autonomic system and its ability to respond to different stimuli (Asgharzadeh-Bonab et al., 2020). The algorithm used for the DFA is described below:

First, the trend of the x series of length N is removed (Peng et al., 1995):

$$y\left(k\right)=\sum_{i=1}^{N}\left(x_{i}-\bar{x}\right)$$ (6)

Then, the detrended series obtained in Equation 6 is segmented into subseries of length *n*, for these subseries the linear fit was obtained by using least squares and the resulting equation *y*_*n*_ *(k)* is subtracted to *y(k)* as follows:

$$F\left(n\right)=\sqrt{\frac{1}{N}\cdot \;\sum_{i=1}^{N}{\left(y\left(k\right)-y_{n}\left(k\right)\right)}^{2}\;}$$ (7)

*F(n)* in Equation 7 measures the fluctuation of the series. This process is repeated for each substring length with $10\leq n\leq \frac{N}{10}$ . Finally, the slope of the linear fit ( γ ) of the $\log_{10}\left(F\left(n\right)\right)$ vs. $\log_{10}\left(n\right)$ graph is obtained.

The γ values were calculated for signals at rest, during exercise, and in the recovery phase, providing a comprehensive view of autonomic regulation under normal and stress conditions.

### 2.8 Multifractal spectrum

It was used a method for direct calculation of the multifractal spectrum proposed by Chhabra and Jensen (Chhabra et al., 1989). The method normalized the time series $P\left(x\right)$ and then it was divided into subseries of length $L=2^{n}$ , after that, the family of normalized measures $\mu_{i}\left(q,L\right)$ was calculated as indicated in Equation 8:

$$\mu_{i}\left(q,L\right)=\frac{{\left[P_{i}\left(L\right)\right]}^{q}}{\sum_{j}{\left[P_{j}\left(L\right)\right]}^{q}}$$ (8)

Then, the fractal dimension $f\left(q\right)$ was calculated by:

$$f\left(q\right)=\lim_{L\to \infty }\frac{\sum_{i}^{N}\left(\mu_{i}\left(q,L\right)\ln\left(\mu_{i}\left(q,L\right)\right)\right)}{\ln\left(L\right)}$$ (9)

Finally, the Hölder exponent $\alpha \left(q\right)$ is obtained from:

$$\alpha \left(q\right)=\lim_{L\to \infty }\frac{\sum_{i}^{N}\left(\mu_{i}\left(q,L\right)\ln\left(P_{i}\left(L\right)\right)\right)}{\ln\left(L\right)}$$ (10)

**FIGURE 1:**
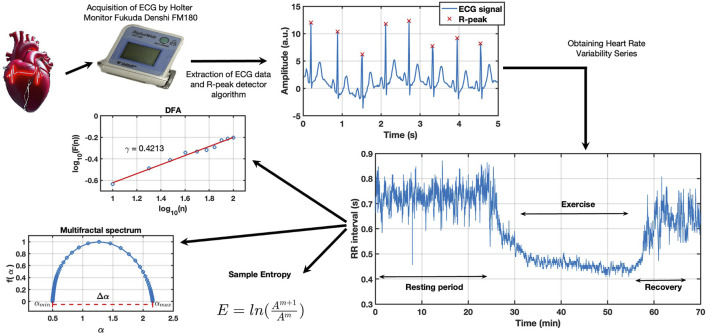
Schematic representation of the workflow for obtaining ECG-derived parameters during rest, exercise, and recovery. The figure illustrates the process from ECG signal acquisition to the extraction of key parameters such as heart rate, entropy, Δα , DFA, recovery time, and post-recovery slope, based on established methodologies.

Equations 9, 10 were follow for each q-value from −30 to 30. The plot formed by $f\left(q\right)$ vs. $\alpha \left(q\right)$ is known as the multifractal spectrum (see Figure 1), and one interesting feature of this graph that can be obtained is the spectrum’s width, which measures the complexity of the series it is known as the multifractality degree, and it is obtained from:

$$\Delta \alpha =\alpha_{\max}-\alpha_{\min}$$ (11)

where $\alpha_{\max}$ and $\alpha_{\min}$ in Equation 11 represents the roots of the spectrum (see Figure 1).

It has been shown that the width of the multifractal spectrum ( Δα ) is larger for healthy and young individuals, and this width decreases with age and even more significantly with disease (Aguilar-Molina et al., 2024).

### 2.9 Principal component analysis (PCA)

A Principal Component Analysis was performed using R software and the FactoMineR and Factoextra packages. This analytical approach allowed for the summarization and visualization of the data sets, describing multiple inter-correlated quantitative variables. Additionally, a concentration ellipse was added around the three study groups present any metabolic alterations (NMS0), had one or two metabolic alterations (NMS2), and with metabolic syndrome by meeting three or more of the ATP III criteria (WSM) (US Department of Health and Human Services, Public Health Service, National Institutes of Health, National Heart, Lung and Blood Institute, and National Cholesterol Education Program, 2001), based on a mean point, using a default confidence level of 0.95 for the underlying Gaussian distribution. The PCA identified the main components (mean variables) that contribute the most to explaining the variance in response patterns among the groups. This analysis was crucial in determining the most relevant parameters that differentiate individuals according to their metabolic profiles.

### 2.10 Comparative statistical analysis

To compare all the most relevant variables that explain the variance and form clusters between the three groups (NMS0, NMS2, WMS) identified by the PCA, a statistical analysis was performed using Welch’s t-test (95% confidence interval) within the R software environment, employing the ggstatplot and ggplot2 packages. Results with a p-value less than 0.05 were considered statistically significant. This analysis allowed for the identification of key differences between the groups, providing a deeper understanding of the variations in metabolic and autonomic responses related to the evaluated parameters.

## 3 Results

### 3.1 Population of study

This study included a total of 278 participants, of which 172 were women and 106 were men. The average age of the population was 28.12 ± 12.5 years, with an age range between 18 and 65 years. In terms of anthropometric characteristics, the average weight of the participants was 66.39 kg, with a minimum weight of 41 kg and a maximum weight of 114.5 kg. The average height was 1.62 m, with a minimum height of 1.4 m and a maximum of 1.85 m. Participants were classified into three groups based on the presence of metabolic alterations, using the ATP III criteria for identifying metabolic syndrome. A total of 93 individuals did not present any metabolic alterations (NMS0), 141 had one or two metabolic alterations (NMS2), and the remaining 44 were diagnosed with metabolic syndrome by meeting three or more of the ATP III criteria.

### 3.2 Detailed analysis of the electrocardiographic signal and autonomic response

The continuous electrocardiogram (ECG) was recorded during the stages of rest, exercise, and recovery, and processed to obtain a series of key parameters reflecting both basic cardiac function and signal variability. Heart rate at rest, during exercise, and in recovery was calculated from the RR intervals in the different phases of the protocol. Resting heart rate was measured during the last 15 min of the initial rest period, heart rate during exercise was recorded over the 30 min of physical activity, and heart rate during recovery was determined during the 15 min following the cessation of exercise.

The sample entropy was calculated over the complete RR time series (it was labeled as E-total), while resting entropy and exercise entropy were specifically calculated for those phases. In addition, Δα and γ values were calculated for complete series (Da-total and g-total, respectively) as long as for resting and exercise series. Δα is a measure of signal complexity, and its decrease reflects a reduction in the range of responses that the heart can provide to stress or external stimuli. On the other hand, γ -values were used to assess long-term correlations in the ECG signal, providing information on the stability of autonomic control. Sample entropy quantifies the complexity and irregularity of heart rate variability time series. Lower sample entropy values indicate more regular and predictable patterns, often associated with aging or pathological conditions, while higher values suggest greater complexity and adaptability of the autonomic nervous system.

Finally, recovery time and post-recovery slope were calculated from the evolution of heart rate after exercise. The recovery time was calculated by truncating the series after exercise until the interbeat interval returned approximately to its original value, and the sum of these RR intervals was computed. On the other hand, the recovery slope was determined using a linear least-squares fitting of the previously truncated series. The recovery slope is smaller in sedentary individuals since they take longer to return to their normal heart rate. In contrast, in healthy individuals who exercise regularly, the slope is steeper, as their heart rate recovers within a few minutes.

### 3.3 Principal component analysis and group representation

**FIGURE 2:**
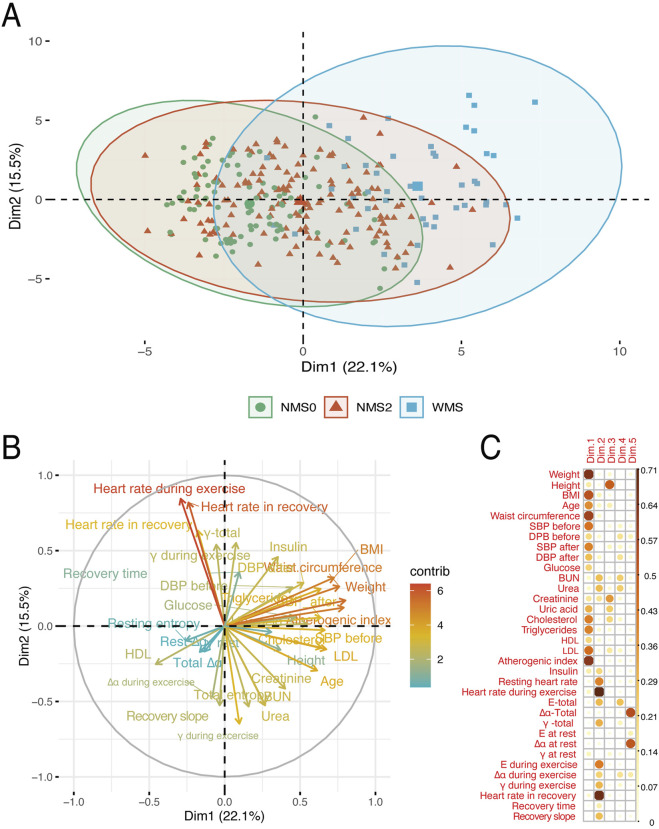
Principal component analysis representing the distribution of individuals across the first two principal components, Dim1 and Dim2, which together explain 37.6% of the total variance (Dim1: 22.1%, Dim2: 15.5%). Individuals without metabolic alterations (NMS0), those with one or two metabolic alterations (NMS2), and those with metabolic syndrome (WSM) are differentiated. **(A)** shows the distribution of individuals in each group with 95% confidence ellipses; **(B)** displays the contribution of each variable to each dimension, with colors indicating the intensity of the contribution; and **(C)** illustrates the correlation of each variable with the principal components in multiple dimensions.

The objective of the PCA was to determine whether could exist a clear separation along the principal components based on the evaluated parameters, which would suggest effective variance between the three groups defined in the study. The analysis revealed that the first two dimensions together explained 37.6% of the total data variance, with 22.1% of the variance explained by the first component and 15.5% by the second (Figure 2).

The first component is strongly influenced by variables related to height-weight and blood pressure, such as body mass index, waist circumference, and systolic blood pressure all of which were recorded at baseline, prior to the exercise protocol (Figure 2). Age was included in the PCA but showed a low contribution to the main components, suggesting that it was not a relevant factor in the separation of groups in this particular sample, which consists predominantly of young adults. Although the influence of age on autonomic function has been well documented in the literature, it did not emerge here as a principal source of variance in our population. Complementary analyses support this decision: the age distribution was centered around young adults, with a mean of was 28.12 ± 12.5 years (Supplementary Figure S1); age also showed only weak correlations with nonlinear ECG-derived variables such as sample entropy and DFA (Supplementary Table S1). Together, these findings indicate that age was not a determining factor in the group separation and therefore was not considered in the subsequent dimensional interpretations. This is reflected in the clear separation of individuals with metabolic syndrome, who cluster in a region with high values on this component, indicating an altered metabolic and cardiovascular profile. In contrast, individuals without metabolic alterations cluster at the opposite end, suggesting a healthier metabolic state. Participants in the NMS2 group occupy an intermediate position, reflecting a moderate metabolic risk.

### 3.4 Individual comparison of relevant parameters

Following the PCA, several parameters were identified as relevant to evidence variance between the study groups. To evaluate the differences in the means of these parameters among the three groups (NMS0, NMS2, and WSM) an individual analysis was performed using Welch’s t-test. This analysis revealed statistically significant differences across a range of parameters, which were divided into two groups based on their clinical relevance and their potential to provide insights into the metabolic and autonomic status of the participants.

**FIGURE 3:**
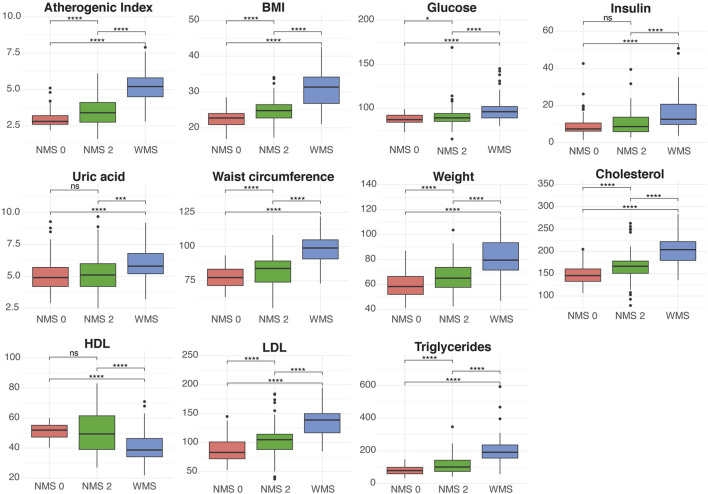
Comparative analysis of traditional metabolic parameters across the three study groups: NMS0 (no metabolic alterations), NMS2 (one or two metabolic alterations), and WSM (with metabolic syndrome). The box plots display the distribution of atherogenic index, BMI, glucose, insulin, uric acid, waist circumference, weight, total cholesterol, HDL, LDL, and triglycerides. Statistical significance was assessed using Welch’s t-test. Significance levels are indicated by asterisks: *p < 0.05, **p < 0.01, ***p < 0.001, ****p < 0.0001, and ns (not significant) for p > 0.05.

The first group includes parameters traditionally associated with the metabolic profile, such as atherogenic index, body mass index (BMI), glucose, insulin, uric acid, waist circumference, weight, total cholesterol, HDL, LDL, and triglycerides (Figure 3). The atherogenic index was higher in the WSM group compared to NMS0 and NMS2, indicating a significant difference in this parameter among the groups. BMI also showed a progressive increase from NMS0 to WSM, with statistically significant differences, suggesting that this parameter increases in relation to the severity of the metabolic profile. Glucose levels were significantly higher in the WSM group compared to the NMS0 and NMS2 groups. Insulin levels were elevated in the WSM group, although the differences between NMS0 and NMS2 were not statistically significant. Uric acid levels were also significantly higher in the WSM group compared to NMS0 and NMS2, indicating a substantial variation in this parameter depending on the metabolic status. Waist circumference showed a notable increase in the WSM group, being significantly larger than in the other groups. Body weight was also significantly higher in the WSM group compared to NMS0 and NMS2. Total cholesterol and LDL levels were higher in the WSM group, with significant differences compared to the other groups, while HDL levels were lower in WSM compared to NMS0 and NMS2. Finally, triglycerides showed significantly elevated levels in the WSM group compared to the other groups, indicating a clear difference in this parameter among the study groups.

**FIGURE 4:**
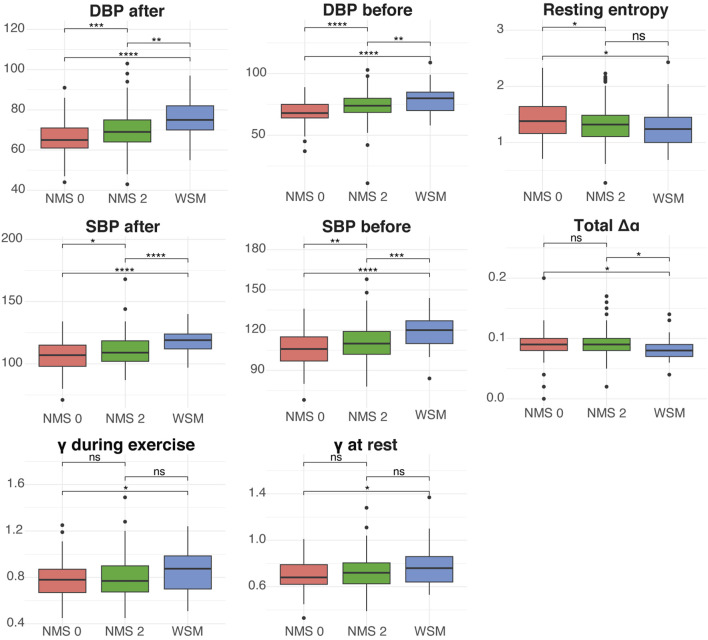
Comparative analysis of parameters derived from exercise and electrocardiogram (ECG) data across the three study groups: NMS0 (no metabolic alterations), NMS2 (one or two metabolic alterations), and WSM (with metabolic syndrome). The box plots display the distribution of diastolic blood pressure (DBP) after exercise, diastolic blood pressure before exercise, resting entropy, systolic blood pressure (SBP) after exercise, systolic blood pressure before exercise, Total Da, DFA during exercise, and DFA at rest. Statistical significance was assessed using Welch’s t-test. Significance levels are indicated by asterisks: *p < 0.05, **p < 0.01, ***p < 0.001, ****p < 0.0001, and ns (not significant) for p > 0.05.

The second group of parameters includes those primarily derived from exercise activity and detailed electrocardiogram (ECG) analysis, such as resting entropy, Δα−Total , γ at rest and during exercise, systolic and diastolic blood pressure before and after exercise, recovery time, and post-recovery slope (Figure 4). Resting entropy was lower in the WSM group compared to NMS0 and NMS2, reflecting lower complexity in the cardiac signal of individuals with metabolic syndrome. Δα−Total also showed differences between the groups, with lower values in the WSM group compared to NMS0, indicating a variation in autonomic regulation based on metabolic status. DFA, both at rest and during exercise, was lower in the WSM group compared to NMS0 and NMS2, with statistically significant differences among the groups, suggesting reduced stability in autonomic control in individuals with metabolic syndrome.

In addition, systolic and diastolic blood pressure measurements before and after exercise showed significant differences among the groups, being higher in the WSM group compared to NMS0 and NMS2.

Finally, recovery time and post-recovery slope showed clear differences between the groups, with longer recovery times and less pronounced slopes in the WSM group, indicating a variation in cardiovascular recovery capacity according to the metabolic profile.

## 4 Discussion

Principal component analysis allowed the identification of key factors that explain a significant portion of the variance between the study groups. Among these factors, resting entropy, heart rate variability, and DFA values were the most relevant for differentiating the NMS0, NMS2, and WSM groups, suggesting that non-traditional markers derived from electrocardiographic analysis may be essential for assessing metabolic risk and identifying autonomic dysfunction. Although the inclusion criteria allowed for a broad age range (18–60 years), the actual distribution of participants was concentrated around young adults due to the nature of voluntary recruitment. As a result, age did not contribute significantly to the PCA variance and was not used for further interpretations. This demographic characteristic should be considered a limitation, and the present findings should not be generalized to older populations without additional studies including more age-diverse groups.

Parameters derived from signal analysis, such as entropy and heart rate variability, are effective in capturing subtle differences in participants’ autonomic and metabolic health and have been suggested as early markers of autonomic deterioration (Carricarte Naranjo et al., 2017; Castiglioni et al., 2022; Ortiz-Guzmán et al., 2023b; Wei et al., 2019). However, our study reveals that decreases in entropy and heart rate variability occur not only in individuals with advanced metabolic syndrome but also in those with one or two metabolic alterations, suggesting that these parameters could serve as early biomarkers of metabolic risk. This suggests that integrating traditional metabolic markers with autonomic parameters provides a more comprehensive view of metabolic health. Although body mass index and blood pressure are critical indicators of metabolic syndrome, the inclusion of autonomic markers such as entropy and DFA adds an additional layer of understanding, especially in the early stages of metabolic dysfunction, allowing for earlier interventions and potentially slowing disease progression.

The findings show notable differences in various traditional metabolic profile parameters between the NMS0, NMS2, and WSM groups. The atherogenic index, BMI, glucose, insulin, uric acid, waist circumference, weight, total cholesterol, HDL, LDL, and triglycerides exhibited progressive alterations as the number of metabolic alterations increased (Figure 3). The WSM group, which includes individuals with metabolic syndrome, exhibited the most pronounced differences in all these parameters compared to the NMS0 and NMS2 groups, evidencing a deteriorated metabolic health state.

It has been extensively demonstrated that BMI and waist circumference are predictors of cardiovascular and metabolic risk, as both are directly related to visceral fat accumulation and insulin resistance (Murguía-Romero et al., 2012). However, recent studies have pointed out the limitations of these indicators when used as sole risk predictors, especially in younger populations or individuals with metabolically healthy obesity (Murguía-Romero et al., 2013). This reinforces the need to consider additional parameters that provide a more accurate picture of metabolic risk (Murguía-Romero et al., 2015).

Moreover, elevated glucose and insulin levels observed in the WSM group align with research (Figure 3), which identifies insulin resistance as a fundamental component of metabolic syndrome (Guerrero-Romero et al., 2016). This phenomenon is a known precursor of type 2 diabetes and other metabolic disorders, emphasizing the importance of monitoring these parameters in patients at risk of progressing to metabolic syndrome.

Another relevant parameter for assessing metabolic risk is uric acid. Although it has been associated with gout and renal dysfunction, uric acid has recently been linked to metabolic alterations, especially in those with metabolic syndrome (Wall-Medrano et al., 2016). Recent findings have shown that elevated uric acid levels may be associated with a higher risk of hypertension, diabetes, and chronic kidney disease (Fahed et al., 2022).

The results show that individuals with metabolic syndrome (WSM group) had significantly higher levels of BMI, waist circumference, plasma lipids (total cholesterol, LDL, triglycerides), glucose, insulin, and uric acid compared to the other groups (Figure 3). The NMS2 group, composed of individuals with one or two metabolic alterations, also showed significant differences in several of these parameters compared to the NMS0 group, particularly in insulin levels, triglycerides, and waist circumference, suggesting that these alterations may be early indicators of insulin resistance and cardiovascular risk (Guerrero-Romero et al., 2016; Wall-Medrano et al., 2016).

It is likely that the combination of traditional and non-traditional markers would enable a more precise and earlier detection of metabolic risk, facilitating preventive interventions in the initial stages (Murguía-Romero et al., 2015; 2012; Srikanthan et al., 2016).

The integration of autonomic parameters with traditional markers provides a more comprehensive view of metabolic health, as demonstrated by the results obtained for systolic (SBP) and diastolic blood pressure (DBP), both before and after exercise. These parameters show a progressive increase from individuals without metabolic alterations (NMS0) to those with one or two alterations (NMS2), and finally, to those with full metabolic syndrome (WSM) (Figure 4). This pattern suggests a close relationship between progressive metabolic deterioration and increased blood pressure, indicating that blood pressure may be a sensitive marker of cardiovascular and metabolic health. The literature has documented that changes in blood pressure are often among the first signs of cardiovascular dysfunction, even before the full manifestation of metabolic syndrome, which underscores the relevance of these findings (Murguía-Romero et al., 2015).

The increase in systolic and diastolic blood pressure in groups with metabolic alterations may also be associated with increased vascular resistance, a phenomenon widely linked to endothelial dysfunction. Endothelial dysfunction is a key factor in the development of cardiovascular diseases and is closely related to insulin resistance, a central component of metabolic syndrome (Wall-Medrano et al., 2016). Recent studies have shown that insulin resistance can lead to increased arterial stiffness and, consequently, higher blood pressure, which aligns with the results observed in this study (Murguía-Romero et al., 2015).

It is important to note that the NMS2 group, which includes individuals with one or two metabolic alterations, showed intermediate blood pressure levels that are significantly higher than those in the NMS0 group (Figure 4). This suggests that blood pressure begins to increase at an early stage of metabolic deterioration, highlighting the importance of monitoring this parameter even in individuals who do not yet meet all criteria for a metabolic syndrome diagnosis. Recent literature has emphasized that early detection of blood pressure changes can be crucial for implementing preventive interventions before irreversible cardiovascular damage occurs (Guerrero-Romero et al., 2016).

The results of detrended fluctuation analysis (DFA) during exercise and at rest reveal significant differences between individuals with metabolic syndrome and those without metabolic alterations, but not between the group with one or two metabolic alterations and the other two groups (Figure 4). These findings suggest that the long-term correlation of the heart rate variability time series, as assessed by DFA, could be a sensitive indicator of fully developed metabolic alterations, though it may be less sensitive to early stages of metabolic syndrome development (Pranata et al., 2017; Sharif et al., 2018; Li, 2020; Nayak et al., 2019).

Our study results also showed a significant reduction in resting entropy in individuals with metabolic syndrome compared to those without metabolic alterations (Figure 4). This finding suggests that the loss of complexity in the heart rate variability time series is associated with impaired autonomic regulation of the cardiovascular system, which is consistent with what has been reported in the literature. The decrease in entropy reflects a reduced capacity of the cardiovascular system to adapt to physiological changes, as observed in several chronic diseases, such as heart failure and other metabolic conditions (Aguilar-Molina et al., 2019). Previous studies have shown that reduced sample entropy is associated with decreased parasympathetic (vagal) activity and lower adaptability of the autonomic nervous system to internal and external stimuli (Costa et al., 2005; Voss et al., 2008). In individuals with metabolic syndrome, this decrease in entropy has been interpreted as a marker of early dysautonomia that precedes overt clinical deterioration (Trivedi et al., 2019). Our findings align with these reports, suggesting that the reduced entropy observed in our MS group reflects an early autonomic inflexibility linked to impaired metabolic control.

Furthermore, we observed a significant decrease in sample entropy in the group of individuals with one or two metabolic alterations (Figure 4), suggesting that the complexity of cardiac signals begins to decline in the early stages of metabolic risk, even before meeting the full criteria for metabolic syndrome. This pattern of early entropy loss has been previously reported, suggesting that autonomic dysfunction could serve as an early marker of metabolic deterioration (Solís-Montufar et al., 2020). Specifically, this dysfunction has been associated with a reduction in parasympathetic modulation and decreased autonomic adaptability, leading to impaired cardiovascular control and reduced flexibility in responding to metabolic demands. These alterations in autonomic tone may precede clinically detectable changes and have been described as early signs of cardiometabolic dysregulation (Ortiz-Guzmán et al., 2023a; Voss et al., 2008).

The literature has consistently shown that young and healthy individuals tend to have higher entropy in their cardiac signals, indicating greater complexity and, therefore, better cardiovascular health. In contrast, individuals with chronic conditions, such as metabolic diseases, exhibit a reduction in entropy, suggesting a dysfunction in autonomic regulation mechanisms (Rojas-Jiménez et al., 2021). This reduction in system complexity reflects a diminished ability to adapt to physiological changes, which has been linked to an increased risk of cardiovascular events in patients with metabolic syndrome and related conditions.

These observations underscore the importance of using non-conventional methods, such as sample entropy, DFA and multifractal spectrum analysis, to capture early alterations in cardiac signals that may not be detected through traditional linear analyses. The ability of sample entropy to detect these early changes reinforces its potential as a promising marker for the early detection of metabolic risk (Muñoz-Diosdado et al., 2023). A methodological limitation of the present study is the absence of standardized dietary control prior to the exercise and recovery phases. Acute food intake may influence metabolic and autonomic responses, potentially introducing variability in HRV measurements.

## 5 Conclusion

Our study shows a significant relationship between reduced sample entropy and the presence of metabolic syndrome, suggesting that entropy could be an important early marker of autonomic dysfunction in individuals at risk of developing this condition. The decrease in dynamic cardiac signal complexity was detectable not only in individuals with fully established metabolic syndrome but also in those with one or two metabolic alterations. This suggests that entropy and other non-traditional markers derived from electrocardiographic analysis, such as DFA, could help identify metabolic dysfunctions before full clinical symptoms emerge, allowing for early intervention and potentially altering the disease trajectory.

Furthermore, connecting these non-traditional markers with conventional metabolic indicators, such as body mass index, blood pressure, and lipid profiles, provides a broader perspective on metabolic health. While traditional indicators are effective for diagnosing metabolic syndrome once it is advanced, the inclusion of parameters such as entropy and DFA enables the detection of subtle deviations in autonomic regulation that may lead to the full manifestation of the condition. This highlights the ability of these markers to enhance risk assessment and support more personalized clinical decisions, focused on preventing the progression toward more severe metabolic syndrome.

## Data Availability

The data is available at the following link: 10.6084/m9.figshare.29296295.v1.
