# Supplementary material for: Utility of nonlinear analysis of heart rate variability in early detection of metabolic syndrome
Source: Front Physiol. 2025 Jun 24;16:1597314. doi: 10.3389/fphys.2025.1597314 (PMC12234528; doi:10.3389/fphys.2025.1597314)
Supplement: Supplementary file 1 [file DataSheet1.docx]

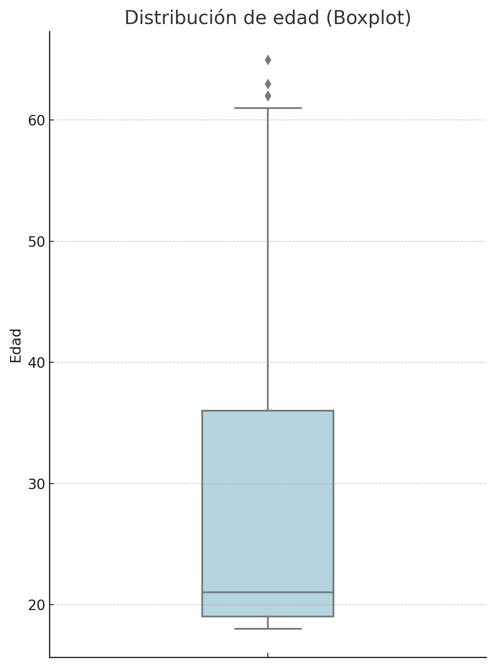


**Supplementary Figure 1**. Distribution of the participants’ age. The boxplot shows the median (central line), the interquartile range (box), and the minimum and maximum values excluding outliers (whiskers).

| Variable | Pearson |
| --- | --- |
| HR_Rest | -0.415 |
| HR_Exercise | -0.487 |
| HR_Recovery | -0.331 |
| Entropy_Total | 0.253 |
| $\Delta\alpha$ _Total | -0.107 |
| $\gamma$ _Total | -0.074 |
| Entropy_Rest | -0.103 |
| $\Delta\alpha$ _Rest | -0.112 |
| $\gamma$ _Rest | 0.35 |
| Entropy_Exercise | 0.193 |
| $\Delta\alpha$_Exercise | 0.056 |
| $\gamma$ _Exercise | -0.035 |

**Supplementary Table 1.** Pearson correlation coefficients between age and selected cardiac autonomic variables, including heart rate, sample entropy, $\gamma$, and $\Delta\alpha$ across rest, exercise, and recovery phases.
